# Global research trends on artificial intelligence in psychological interventions for stroke survivors: a bibliometric and visualized analysis (2000–2024)

**DOI:** 10.3389/fpsyg.2025.1541092

**Published:** 2025-06-26

**Authors:** Yaoyao Li, Kim Lam Soh, Fengna Sun, Lili Wei, Hasni Idayu Saidi, Kim Geok Soh

**Affiliations:** ^1^Department of Nursing, Faculty of Medicine and Health Sciences, Universiti Putra Malaysia, Serdang, Selangor, Malaysia; ^2^Department of Geriatrics, Zibo Central Hospital, Zibo, Shandong, China; ^3^Department of Dean’s Office, The Affiliated Hospital of Qingdao University, Qingdao, Shandong, China; ^4^Department of Biomedical Science, Faculty of Medicine and Health Sciences, Universiti Putra Malaysia, Serdang, Selangor, Malaysia; ^5^Department of Sports Studies, Faculty of Educational Studies, Universiti Putra Malaysia, Serdang, Selangor, Malaysia

**Keywords:** artificial intelligence, bibliometric analysis, stroke survivors, psychological intervention, CiteSpace, VOSviewer

## Abstract

**Objective:**

This study aimed to conduct a bibliometric analysis of research literature on AI-assisted psychological interventions for stroke survivors published from 2000 to 2024, using CiteSpace and VOSviewer to examine research collaboration networks, knowledge structures, and developmental trends.

**Methods:**

Literature data was sourced from the Web of Science Core Collection database (WoSCC). A total of 450 relevant articles, published between 1 January 2000 and 31 October 2024, were included. CiteSpace and VOSviewer were employed to analyze the literature from dimensions including authorship, country of origin, institutions, journals, references, and keywords.

**Results:**

Annual publication output has steadily increased, reaching a peak in 2022 (72 articles), followed by 68 articles in 2023 and 69 articles in 2024. Calabro Rocco Salvatore emerged as the most productive author (9 publications). The United States led in research output (87 articles) and centrality (0.51), with McGill University being the leading institution (10 articles). “Journal of NeuroEngineering and Rehabilitation” was the most productive journal (24 articles), while “Stroke” was the most co-cited journal (347 times). Recent research trends focused on ischemic stroke (strength = 3.09), anxiety (strength = 2.72), cognitive impairment (strength = 2.67), and meta-analysis (strength = 2.58), reflecting a shift toward integrated assessment and intervention strategies.

**Conclusion:**

This bibliometric analysis reveals a significant evolution in research on AI-assisted psychological interventions for stroke survivors, shifting from single technology applications to integrated services encompassing assessment, monitoring and intervention. Future research should continue to strengthen empirical studies while enhancing interdisciplinary collaboration to improve the quality of psychological healthcare services.

## 1 Introduction

Stroke, as the second leading cause of mortality and primary disabling factor worldwide, poses a serious threat to human health. According to recent studies, approximately 13 million new stroke cases occur globally each year, with one-third of patients developing permanent functional disabilities (Thayabaranathan et al., 2022). Clinical evidence indicates that stroke survivors face not only physical manifestations including motor dysfunction and speech impairments but also complex mental health challenges. Recent systematic reviews indicate that approximately 45–65% of patients present with depressive symptoms, while 35–55% manifest anxiety symptoms (Curatoli et al., 2023; Jacobs and Ellis, 2023). Additionally, approximately 20–30% of patients develop post-traumatic stress disorder and adjustment disorders (Oei et al., 2023; Tjokrowijoto et al., 2024). These mental health complications significantly influence patients’ rehabilitation adherence and quality of life, ultimately leading to higher mortality and disability rates (Chen et al., 2020; Tsai et al., 2023).

With advances in science and technology, particularly artificial intelligence developments since 2000, new solutions have been provided for mental health interventions among stroke survivors. Specifically, intelligent applications based on machine learning, natural language processing and computer vision technologies have not only overcome the spatial and temporal limitations of traditional psychological interventions but also provided technical support for personalized and precise mental health services (Shek et al., 2021). This innovation stems from researchers’ in-depth understanding of patients’ mental health needs and the unique advantages demonstrated by artificial intelligence in chronic disease management (Mou and Wong, 2021). Research indicates that AI-based intelligent intervention systems can provide continuous mental health monitoring, identifying potential mental health risks through analyzing patients’ behavioral patterns and emotional changes (Hinwood et al., 2020). Randomized controlled trials further confirm that AI-assisted psychological interventions significantly improved patients’ rehabilitation adherence (by 35.6%) and therapeutic engagement (by 42.3%), while enhancing patients’ social functioning and quality of life scores (by 28.7%) (Han, 2023). Moreover, intelligent psychological interventions incorporating natural language processing technology can automatically adjust intervention strategies based on patients’ real-time feedback, resulting in a 40.2% improvement in intervention effectiveness (Chin et al., 2022).

Despite the rapid growth in related research, there remains a lack of comprehensive analysis regarding the overall development trajectory of artificial intelligence in psychological interventions for stroke survivors. This field involves the cross-integration of multiple disciplines, including artificial intelligence, psychology, and rehabilitation medicine, featuring complex knowledge structures and rapid developmental dynamics (Guo et al., 2020). Although traditional literature reviews have provided important references for understanding field development, they demonstrate certain limitations in revealing multidisciplinary characteristics, technological evolution pathways, and knowledge mapping (Tran et al., 2019). Bibliometrics, as a data-based objective analysis method, can reveal the evolution patterns of research hotspots, interdisciplinary characteristics, and collaboration network features through multidimensional quantitative analysis of literature metrics, citation relationships, and knowledge structures (Liu et al., 2022). Particularly in rapidly developing emerging fields such as artificial intelligence, bibliometrics can not only clearly demonstrate the evolution trajectory of technological applications but also predict future development trends through knowledge mapping analysis, providing reference evidence for field development (Nawi et al., 2023; Choo et al., 2023).

Therefore, this study aims to utilize bibliometric methods, based on the Web of Science Core Collection (WoSCC), to analyze research literature on AI-assisted psychological interventions for stroke survivors published between 2000 and 2024, utilizing CiteSpace and VOSviewer software to examine research development trends from multiple dimensions such as author collaboration, country/institutional distribution, journal distribution, references and keywords. By revealing the evolution of research hotspots, knowledge structure characteristics and development momentum in this field over the past 25 years, this study provides scientific evidence to promote the in-depth development of artificial intelligence technology in psychological interventions for stroke survivors. Meanwhile, this study expects to provide references for future research directions and practical innovations through bibliometric analysis of research frontiers in this field.

## 2 Methods

### 2.1 Search strategy

This study relied on the Web of Science Core Collection (WoSCC) to retrieve relevant literature. As a widely recognized high-quality digital literature database, WoSCC is frequently considered the optimal resource for conducting bibliometric analyses (Guo et al., 2020). The Science Citation Index (SCI) Expanded (1900-present) was selected as the retrieval citation index to guarantee the thoroughness and accuracy of the collected data (Liu, 2021; Ho, 2020).

The retrieval strategy is as follows:

Topic 1(TS) = (“stroke survivor*” OR “post-stroke” OR “poststroke” OR “stroke patient*” OR “cerebrovascular accident*” OR “CVA” OR “stroke rehabilitation” OR “stroke victim*” OR “stroke suffer*”) AND
Topic 2(TS) = (“artificial intelligence” OR “AI” OR “machine learning” OR “deep learning” OR “neural network*” OR “natural language processing” OR “NLP” OR “emotion recognition” OR “sentiment analysis” OR “virtual reality” OR “VR” OR “digital health” OR “digital therapeutics” OR “intelligent system*” OR “chatbot*” OR “conversational agent*” OR “augmented reality” OR “AR” OR “mixed reality” OR “automated intervention*” OR “digital intervention*”) AND
Topic 3(TS) = (“psychological intervention*” OR “mental health” OR “psychological well-being” OR “well-being” OR “psychological resilience” OR “emotional support” OR “psychotherapy” OR “cognitive behavior* therapy” OR “CBT” OR “psychosocial intervention*” OR “mental health intervention*” OR “mindfulness” OR “self-efficacy” OR “quality of life” OR “QoL” OR “depression” OR “anxiety” OR “emotional rehabilitation” OR “psychological distress” OR “mood disorder*” OR “psychological adaptation” OR “mental well-being” OR “psychological support” OR “emotional adjustment” OR “psychological rehabilitation” OR “mental healthcare” OR “stress” OR “psychological status”).

The retrieval timeframe was set from 1 January 2000 to 31 October 2024, to comprehensively reflect the latest developments of artificial intelligence in psychological intervention research for stroke survivors. To reflect both original research outcomes and summary perspectives in this field, the literature types included for analysis primarily comprised research articles and review articles. Considering English as the primary language in international research, we limited the literature language to English to ensure the readability of included articles and the generalisability of research findings.

#### 2.1.1 Inclusion criteria

Inclusion criteria were: (i) original research articles and review articles; (ii) articles published between January 1, 2000 and October 31, 2024; (iii) articles written in English; (iv) articles simultaneously addressing stroke survivors/patients, artificial intelligence applications, and psychological interventions; (v) articles taken from the WoSCC database.

#### 2.1.2 Exclusion criteria

The following were the exclusion criteria: (i) editorials, conference abstracts, book reviews, newsletters, and letters; (ii) duplicate publications or multiple reports from the same study; (iii) articles with inaccessible full text.

Following the database retrieval, manual screening was conducted by two independent researchers (YYL and KLS) to ensure that all included articles were relevant to AI-assisted psychological interventions for stroke survivors. Any disagreements were resolved through discussion with a third researcher until consensus was reached (LLW).

### 2.2 Analysis tools

This study utilizes multiple bibliometric analysis tools and visualization software to comprehensively analyze the bibliometric characteristics of research on AI-assisted psychological interventions for stroke survivors. Initially, Microsoft Excel 2019 was used for preliminary statistical analysis of the original literature data, including basic characteristics such as annual literature distribution and literature type composition (Guo et al., 2020). Subsequently, CiteSpace 6.2.3 R3 and VOSviewer 1.6.17 software were used for in-depth bibliometric analysis and visualization. Both software programs are developed on the Java platform and are widely used information visualization tools in bibliometric research (Tu et al., 2022). This methodological combination enables the identification of collaboration networks, emerging research trends, and keyword clusters in an efficient and visually appealing manner (Yang et al., 2022). For co-citation analyses, thresholds were primarily set at 30 citations based on established bibliometric practices (Chen, 2017), while selective adjustments to higher or lower thresholds were applied in specific visualizations as documented in respective result sections to balance comprehensive data representation and visualization clarity (Chen, 2017).

Utilizing CiteSpace software, we performed multidimensional analysis of the literature data on AI-assisted psychological interventions for stroke survivors. Firstly, we generated an author publication network map to identify the core authors and their publication volumes in this field. Based on this, we performed cluster analysis of the author network to explore research teams and collaborative groups in this field. Additionally, we generated author co-citation network maps to identify scholars with significant influence in this field. Through CiteSpace, we also created country and institutional maps to demonstrate the geographical distribution characteristics and institutional collaboration patterns in this field. In these network analyses, we employed the betweenness centralities to measure each node’s importance within the collaboration network. This indicator, ranging from 0 to 1, calculates how frequently a node lies on the shortest paths between other node pairs, with values above 0.1 considered significant. Higher centrality values indicate greater roles in facilitating information flow and knowledge transfer across the research network. Meanwhile, we conducted dual-map overlay analysis of journals to illustrate the interdisciplinary knowledge exchange and dissemination in this field.

**Figure 1:**
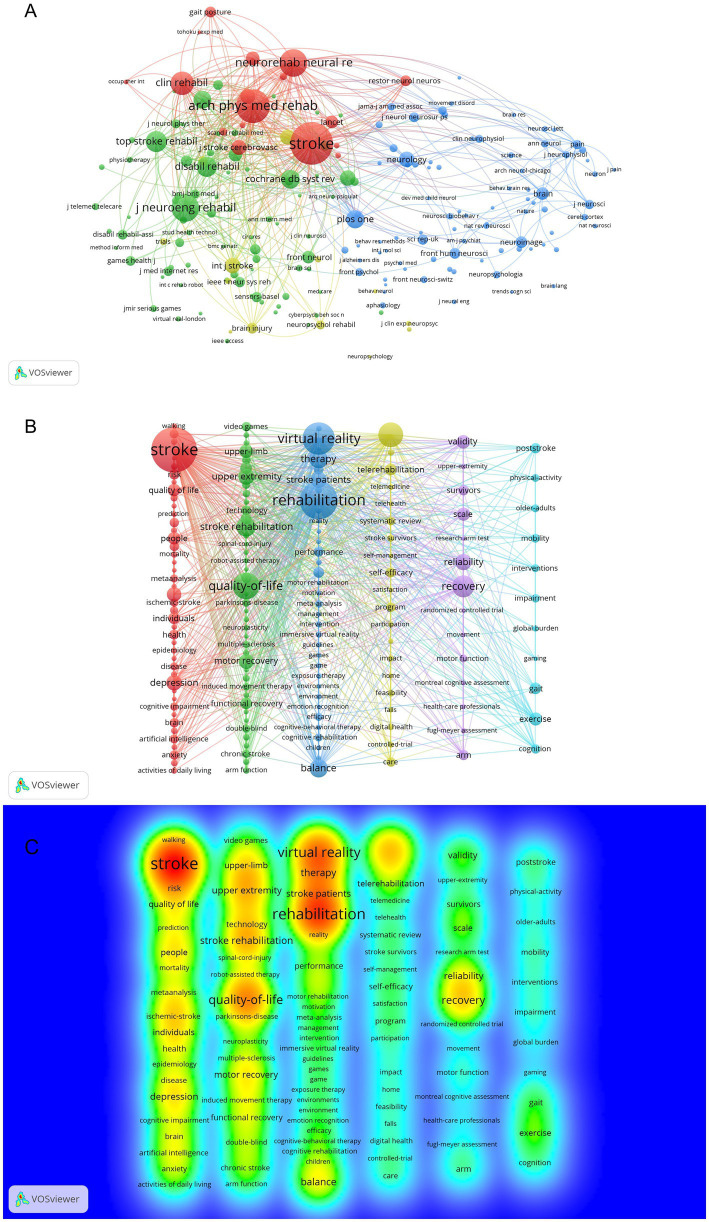
Bibliometric visualization maps for AI-assisted psychological interventions in stroke survivors. **(A)** Map of co-cited journals with publications. **(B)** Map of co-occurring keywords with occurrences. **(C)** Map of keywords density.

Using VOSviewer, we conducted analysis of the knowledge structure and research theme evolution in AI-assisted psychological interventions for stroke survivors. By generating journal co-citation networks based on publication volumes (Figure 1A), we identified the significant journals and their citation patterns in this field. Through keyword co-occurrence analysis, we constructed keyword co-occurrence networks to highlight the structural distribution of research themes and hotspots (Figure 1B). Meanwhile, using keyword density visualization maps, we intuitively demonstrated the knowledge density and distribution of research themes (Figure 1C). Additionally, we conducted visualization analysis of country/region distribution in the literature data to reveal the global research landscape in this field. Finally, by generating reference co-citation networks, we identified the knowledge context and theoretical foundations in this field.

### 2.3 Research ethics

This study was conducted as a bibliometric analysis. All data were sourced from online resources, and no animals or human subjects were involved. Therefore, ethical approval from an ethics committee was not required.

## 3 Results

### 3.1 Analysis of publication output and citations

**Figure 2:**
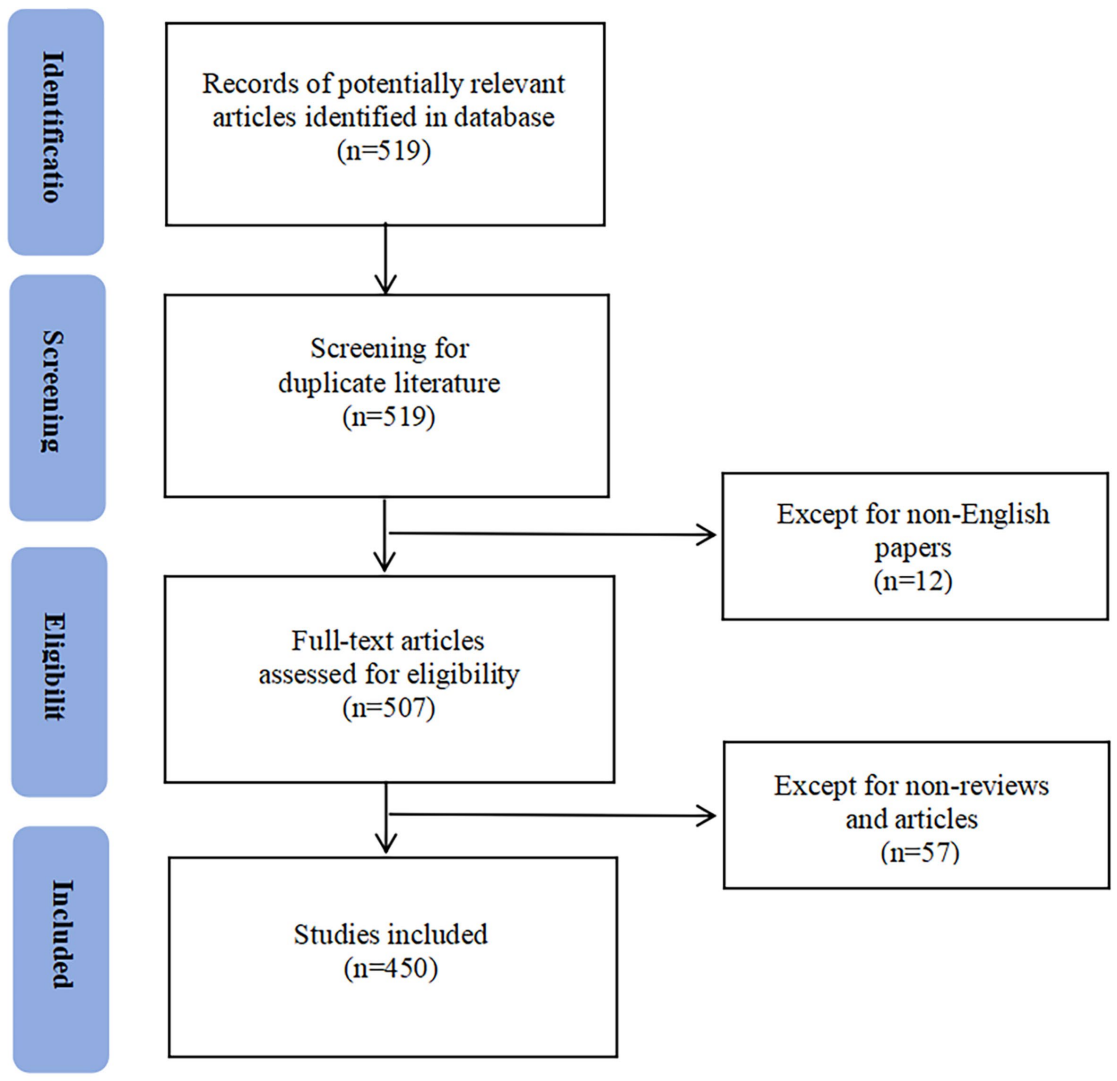
The flow chart of screening process.

**Figure 3:**
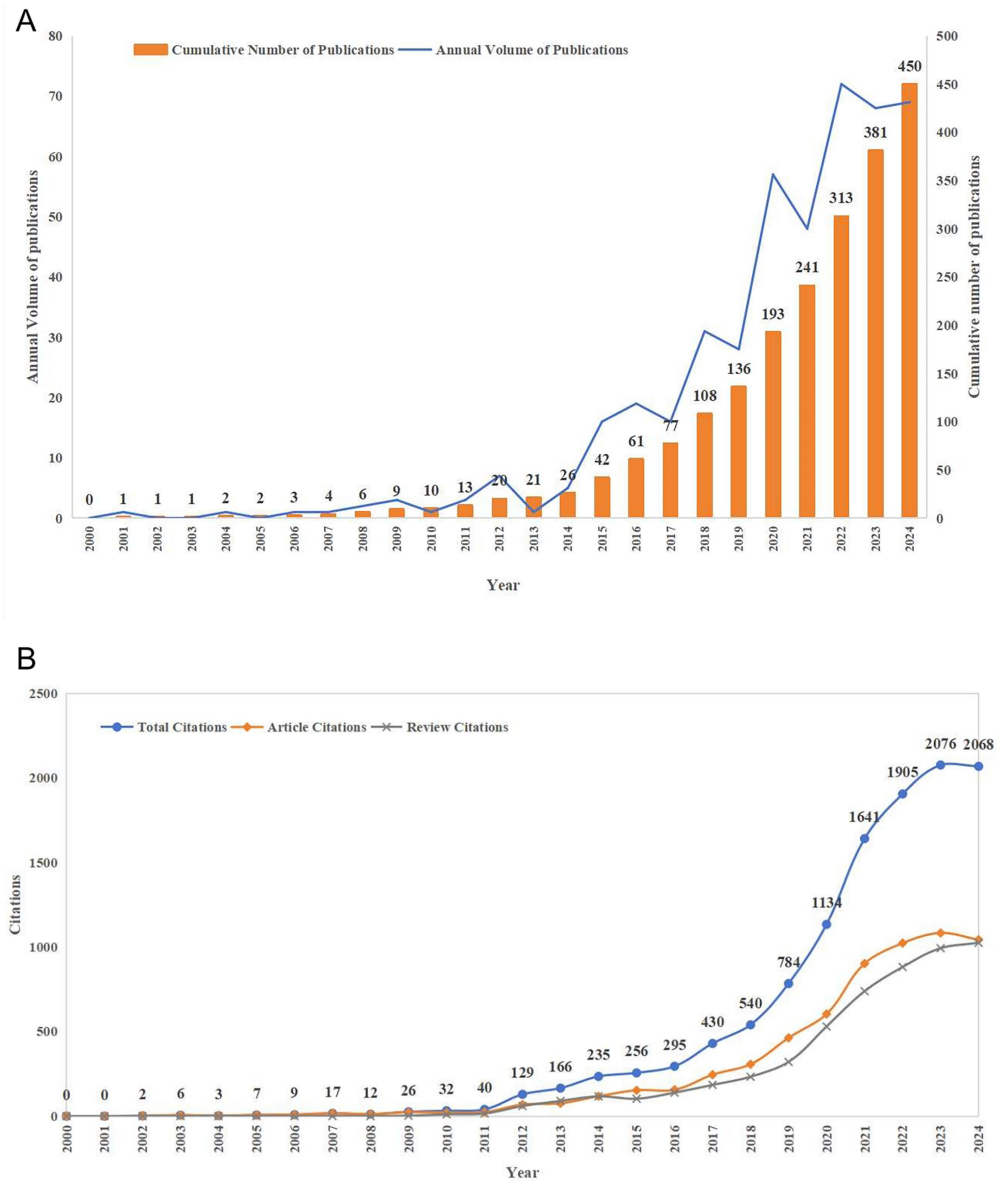
Publications and citations analysis for AI-assisted psychological interventions in stroke survivors. **(A)** The publication output and its growth trend from 2000 to 2024. **(B)** The annual citation count from 2000 to 2024.

From 1 January 2000 to 31 October 2024, the Web of Science Core Collection database yielded a total of 450 articles that met the inclusion criteria, comprising 330 research articles and 120 review articles (Figure 2). Publications analysis showed that 2022 was the year with the highest publication output, reaching 72 articles, while the field’s scholarly output has shown a steady increasing trend since 2015 (Figure 3A). Citations analysis revealed that these 450 articles have been cited a total of 11,813 times, with 2023 recording the highest number of citations at 2,076 (Figure 3B). Most publications appeared within the past decade, indicating that AI-assisted psychological interventions for stroke survivors is a rapidly evolving field that is attracting increasing attention from researchers.

### 3.2 Analysis of authors and co-cited authors

**Figure 4:**
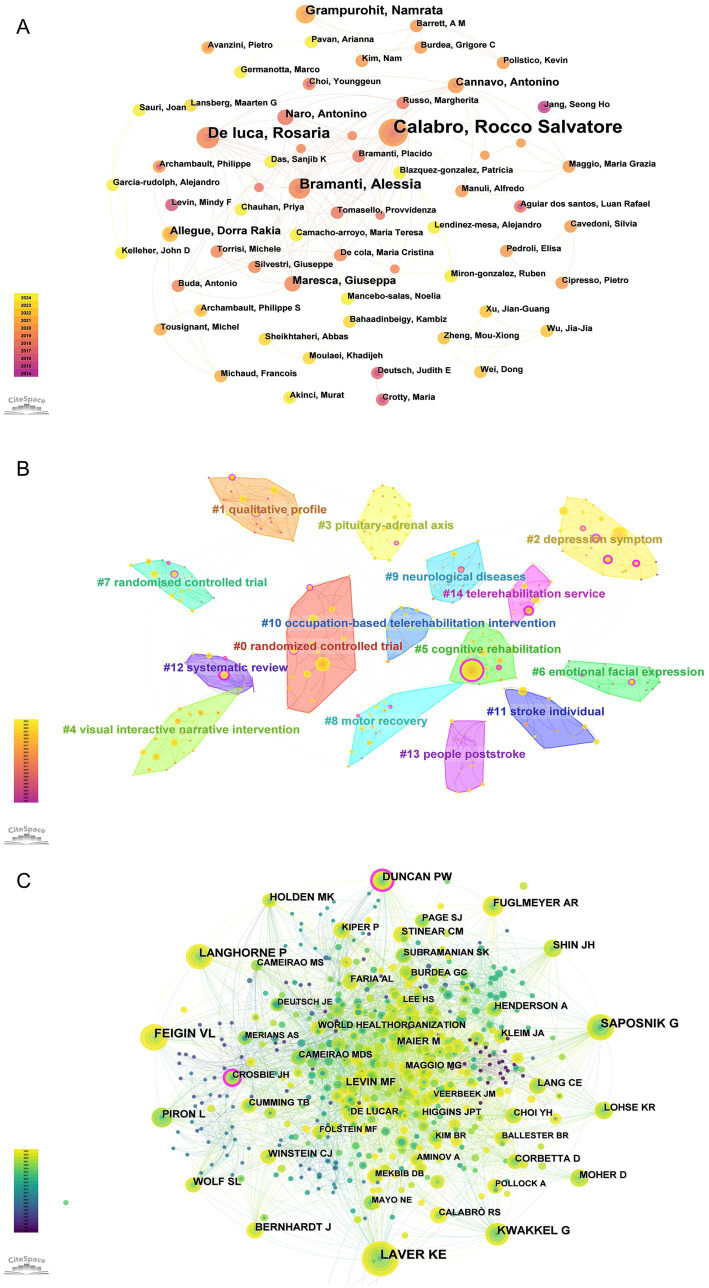
Author network analysis for AI-assisted psychological interventions in stroke survivors. **(A)** Map of authors with publications. **(B)** Map of clustering of authors. **(C)** Map of co-cited authors with publications.

Using CiteSpace with a threshold of 30, author collaboration network analysis revealed that 2,605 authors contributed to the 450 articles, with Calabro Rocco Salvatore emerging as the most productive author (9 publications) (Figure 4A; Table 1). Cluster analysis identified 15 major research themes including cognitive rehabilitation, emotional expression, motor rehabilitation services, and telerehabilitation interventions (Figure 4B). Co-citation analysis showed Laver KE had the highest citation count (115), while Crosbie JH showed the highest centrality (0.18) (Figure 4C; Table 1). These researchers have significantly contributed to advancing both theoretical understanding and practical applications in AI-assisted psychological interventions for stroke survivors.

**Table 1:** The top five authors and co-cited authors about AI-assisted psychological interventions for stroke survivors.

Rank	Authors	Count	Cited authors	Count times	Cited authors	Centrality
1	Calabro, Rocco Salvatore	9	Laver KE	115	Crosbie JH	0.18
2	De luca, Rosaria	6	Feigin VL	85	Duncan PW	0.13
3	Bramanti, Alessia	5	Saposnik G	78	Laver KE	0.09
4	Deutsch, Judith E	4	Langhorne P	68	Cameirao MS	0.09
5	Grampurohit, Namrata	4	Kwakkel G	64	Kwakkel G	0.08

### 3.3 Analysis of countries and institutions

**Figure 5:**
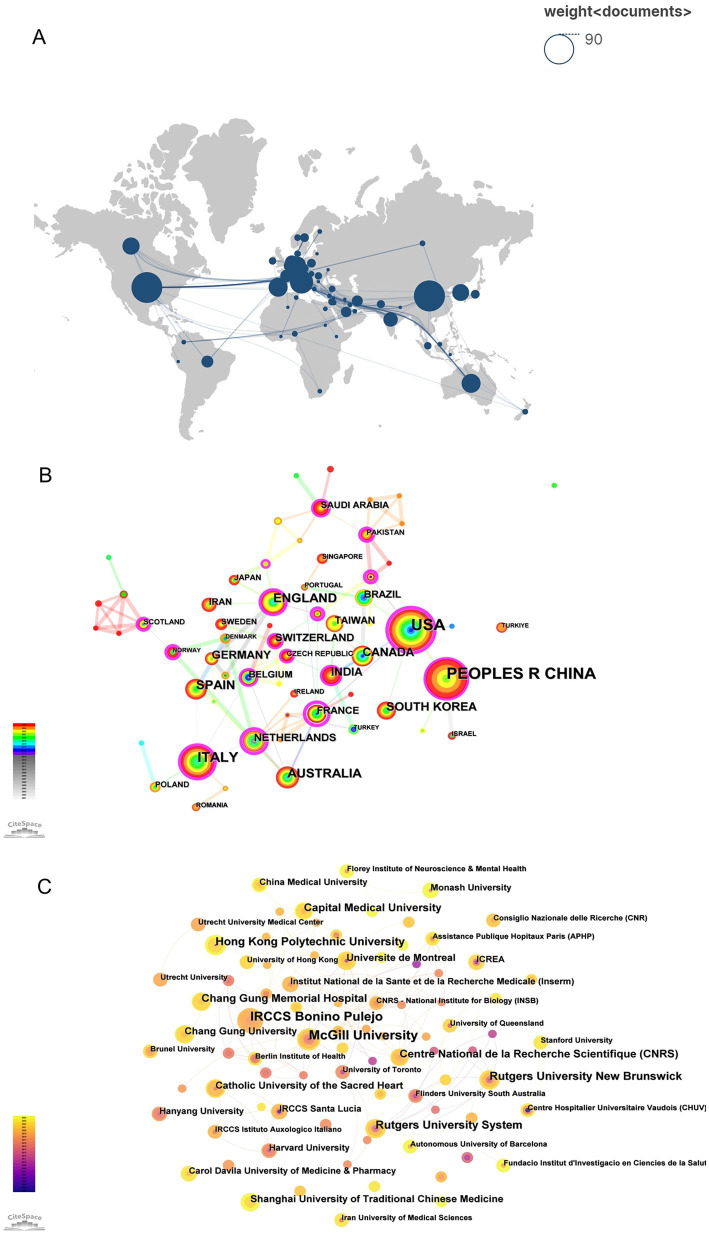
**(A)** Geographic distribution of countries/regions in AI-assisted psychological interventions for stroke survivors. **(B)** Map of countries in AI-assisted psychological interventions for stroke survivors. **(C)** Map of institutions in AI-assisted psychological interventions for stroke survivors.

Publications in this research field originated from 65 countries/regions (Figure 5A). The top five countries contributed over 61% of the publications. To explore international collaboration networks, we analyzed the relationships between publications from different countries using CiteSpace with a threshold of 5, which identified research contributions from 65 countries/regions (Figure 5B). Results showed that the United States had the highest number of publications (87), followed by China with 76 publications and Italy with 49 publications, with Australia and Spain contributing 33 publications each (Table 2). For centrality analysis, France showed the highest centrality (0.64), followed by the Netherlands (0.54), the United States (0.51), Russia (0.48) and New Zealand (0.41). These countries played key influential roles in global research collaboration.

**Table 2:** The top five countries in terms of count and centrality in AI-assisted psychological interventions for stroke survivors.

Rank	Country	Count	Country	Centrality
1	USA	87	France	0.64
2	Peoples R China	76	Netherlands	0.54
3	Italy	49	USA	0.51
4	Spain	33	Russia	0.48
5	Australia	33	New Zealand	0.41

At the institutional level, we selected ‘institution’ as the node to generate an institutional distribution map (Figure 5C), applying a minimum threshold of 3 to focus on significant institutions. Results revealed the top five most productive institutions in this research field, McGill University had the highest number of publications (10), followed by IRCCS Bonino Pulejo (9 publications), with Rutgers University System, Hong Kong Polytechnic University and Rutgers University New Brunswick (7 publications each). Centrality analysis identified that McGill University, University of Toronto, CNRS - National Institute for Biology (INSB) and FHNW University of Applied Sciences & Arts Northwestern Switzerland had the highest centrality (0.02), followed by Flinders University South Australia (0.01) (Table 3).

**Table 3:** The top five institutions in terms of count and centrality in AI-assisted psychological interventions for stroke survivors.

Rank	Institution	Count	Institution	Centrality
1	McGill University	10	McGill University	0.02
2	IRCCS Bonino Pulejo	9	University of Toronto	0.02
3	Rutgers University System	7	CNRS - National Institute for Biology (INSB)	0.02
4	Hong Kong Polytechnic University	7	FHNW University of Applied Sciences & Arts Northwestern Switzerland	0.02
5	Rutgers University New Brunswick	7	Flinders University South Australia	0.01

### 3.4 Analysis of journals and co-cited journals

**Figure 6:**
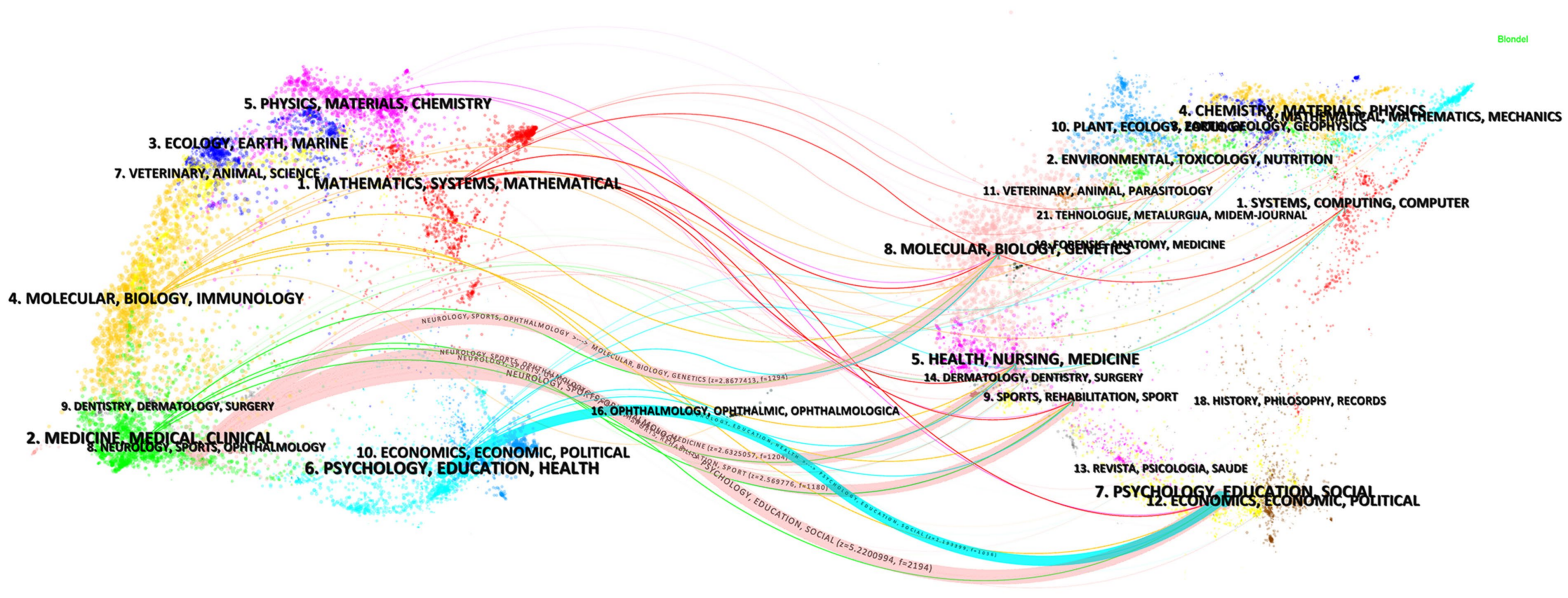
The dual-map overlay of journals about AI-assisted psychological interventions for stroke survivors.

Based on 226 journals analyzed, dual-map overlay analysis revealed that literature from neurology, sports and ophthalmology fields was mainly influenced by literature from psychology, education and social fields (*z* = 5.22, *f* = 2,194), and literature from molecular biology and genetics fields (*z* = 2.87, *f* = 1,294) (Figure 6). Analysis of journal publications showed Journal of NeuroEngineering and Rehabilitation had the highest number of publications (24 articles), followed by Frontiers in Neurology (17 articles) (Table 4). Journal co-citation analysis revealed distinct clusters of journals in stroke rehabilitation, intelligent rehabilitation technologies, and AI applications in emotion recognition (Figure 1A). Stroke had the highest co-citation count (347), while Brain Injury had the highest centrality (0.13) (Table 5). Results indicate the multidisciplinary foundation of the research field.

**Table 4:** The top 10 journals by publications about AI-assisted psychological interventions for stroke survivors.

Rank	Source (Abbreviations)	Publications	Citations	Average citations/ publications	Country	IF (2023)
1	Journal of NeuroEngineering and Rehabilitation (J Neuroeng Rehabil)	24	1,129	47.04	England	5.2
2	Frontiers in Neurology (Front Neurol)	17	143	8.41	Switzerland	2.7
3	Journal of Stroke & Cerebrovascular Diseases (J Stroke Cerebrovasc Dis)	11	450	40.91	United States	2.0
4	International Journal of Environmental Research and Public Health (Int J Env Res Pub He)	10	188	18.80	Switzerland	4.6
5	Journal of Medical Internet Research (J Med Internet Res)	9	211	23.44	Canada	5.8
6	Topics in Stroke Rehabilitation (Top Stroke Rehabil)	9	300	33.33	United States	2.2
7	BMJ Open	8	63	7.88	England	2.4
8	JMIR Serious Games	8	180	22.50	Canada	3.8
9	Sensors (Sensors-Basel)	8	133	16.63	Switzerland	3.4
10	Cochrane Database of Systematic Reviews (Cochrane Db Syst Rev)	7	1,461	208.71	England	8.1

**Table 5:** The top five co-cited journals by counts and centrality about AI-assisted psychological interventions for stroke survivors.

Rank	Co-cited counts	Cited journal (Abbreviations)	Centrality	Cited journal (Abbreviations)
1	347	Stroke	0.13	Brain Injury
2	290	Archives Of Physical Medicine And Rehabilitation (Arch Phys Med Rehab)	0.11	Acta Neurologica Scandinavica (Acta Neurol Scand)
3	227	Journal of NeuroEngineering and Rehabilitation (J Neuroeng Rehabil)	0.08	Archives Of Physical Medicine And Rehabilitation (Arch Phys Med Rehab)
4	214	Topics In Stroke Rehabilitation (Top Stroke Rehabil)	0.08	American Journal of Physical Medicine & Rehabilitation (Am J Phys Med Rehab)
5	211	Neurorehabilitation And Neural Repair (Neurorehab Neural Re)	0.08	Aging Research Reviews (Aging Res Rev)

### 3.5 Analysis of co-cited references

**Figure 7:**
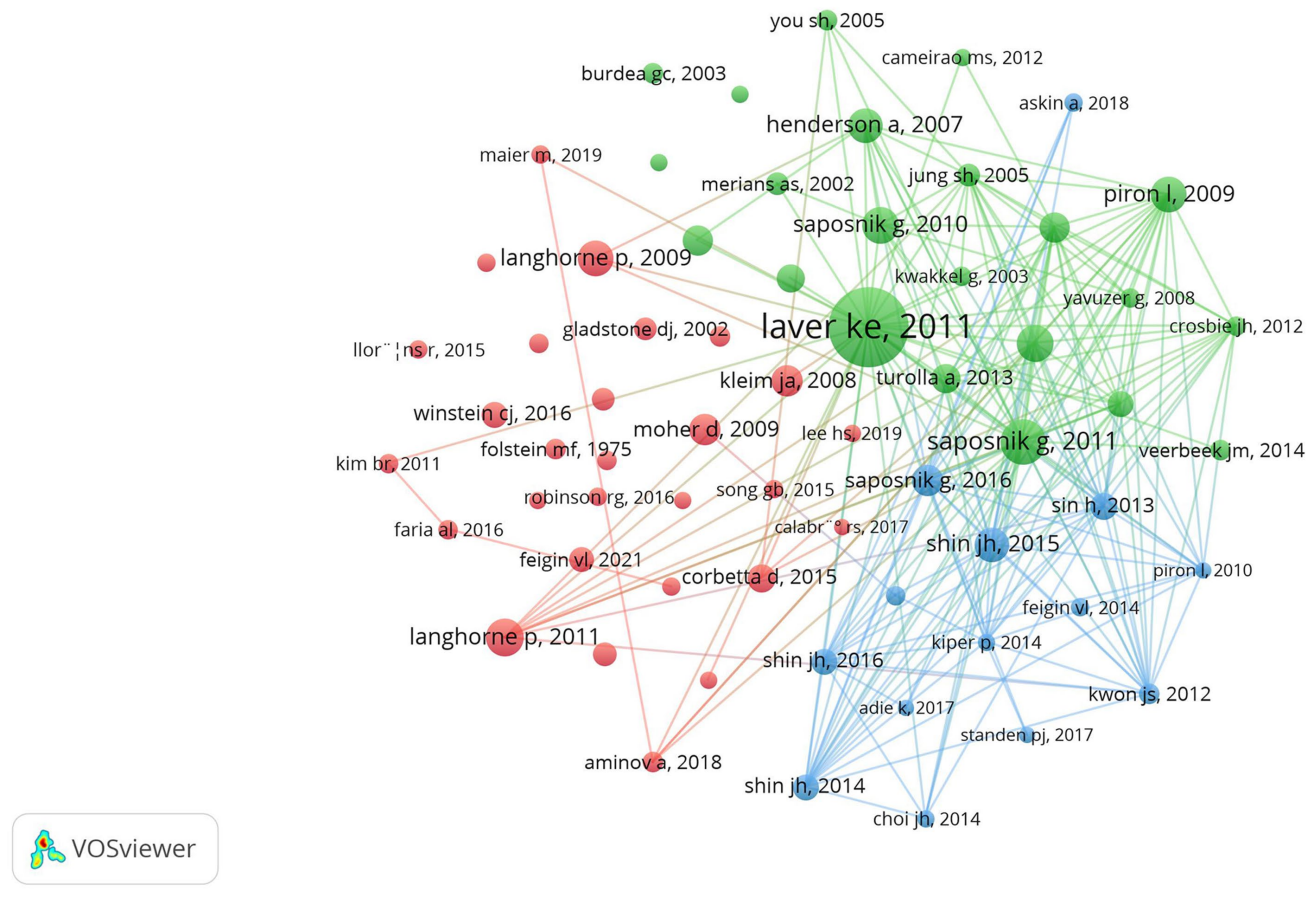
Map of co-cited references with citations in AI-assisted psychological interventions for stroke survivors.

Reference co-citation analysis was conducted using VOSviewer on 22,175 references, with 65 core references meeting the minimum threshold of 15 co-citations (Figure 7). Analysis revealed the top 10 most cited references, including five research articles and five review articles published between 2007 and 2016, with “Virtual reality for stroke rehabilitation” by Laver et al. (2011) having the highest citation count (71 citations) (Table 6). The co-citation network identified three distinct research clusters focusing on evidence-based stroke rehabilitation, virtual reality applications, and intelligent rehabilitation system development (Figure 7).

**Table 6:** The top 10 co-cited references about AI-assisted psychological interventions for stroke survivors.

Rank	Title	Citations	Year	First author	Journal	Document type
1	Virtual reality for stroke rehabilitation	71	2011	Laver K E	Cochrane Database of Systematic Reviews	Review
2	Virtual reality in stroke rehabilitation: a meta-analysis and implications for clinicians	40	2011	Saposnik G	Stroke	Review
3	Stroke rehabilitation	34	2011	Langhorne P	The Lancet	Research
4	Virtual reality therapy for adults post-stroke: a systematic review and meta-analysis exploring virtual environments and commercial games in therapy	33	2014	Lohse K R	PloS one	Review
5	Effectiveness of virtual reality using Wii gaming technology in stroke rehabilitation: a pilot randomized clinical trial and proof of principle	33	2010	Saposnik G	Stroke	Research
6	Motor recovery after stroke: a systematic review	32	2009	Langhorne P	The Lancet Neurolog	Review
7	Exercises for paretic upper limb after stroke: a combined virtual-reality and telemedicine approach	32	2009	Piron L	Journal of rehabilitation medicine	Research
8	Virtual reality in stroke rehabilitation: a systematic review of its effectiveness for upper limb motor recovery	31	2007	Henderson A	Topics in stroke rehabilitation	Review
9	Effects of game-based virtual reality on health-related quality of life in chronic stroke patients: a randomized, controlled study	31	2015	Shin J H	Computers in biology and medicine	Research
10	Efficacy and safety of non-immersive virtual reality exercising in stroke rehabilitation (EVREST): a randomized, multicentre, single-blind, controlled trial	28	2016	Saposnik G	The Lancet Neurology	Research

### 3.6 Analysis of keywords

As of October 2024, a total of 2,161 keywords were identified in this research field. Using VOSviewer software, we set a minimum co-occurrence frequency of 6, with 165 keywords meeting this threshold for analysis (Figure 1B). The keyword network analysis revealed six clusters representing psychological health, virtual reality interventions, rehabilitation strategies, telerehabilitation, rehabilitation outcomes, and population-specific interventions. Meanwhile, to provide a more intuitive representation of research hotspots, we generated a density visualization map of keywords (Figure 1C), where warmer colors indicate higher research focus. Additionally, to reveal the temporal evolution of research hotspots, we conducted strongest citation bursts analysis of keywords (Table 7). Recent research trends focused on ischemic stroke (strength = 3.09), anxiety (strength = 2.72), and cognitive impairment (strength = 2.67), with burst periods from 2022 to 2024.

**Table 7:** The top 20 keywords with citation bursts about AI-assisted psychological interventions for stroke survivors.

Keywords	Year	Strength	Begin	End	2000–2024
brain injury	2006	2.56	2006	2016	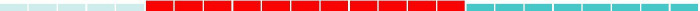
traumatic brain injury	2008	3.83	2008	2017	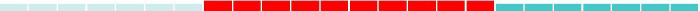
environment	2009	2.41	2009	2015	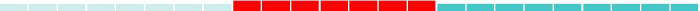
randomized controlled trial	2015	3.31	2015	2017	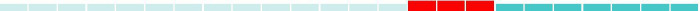
virtual reality therapy	2016	3.67	2016	2017	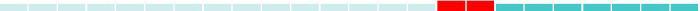
controlled trial	2016	3.54	2016	2019	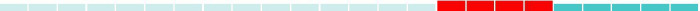
stroke patients	2004	2.25	2017	2018	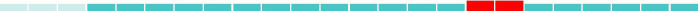
individuals	2011	3.69	2018	2020	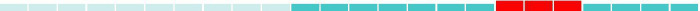
transcranial magnetic stimulation	2019	2.74	2019	2020	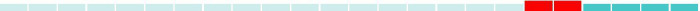
occupational therapy	2019	2.49	2019	2021	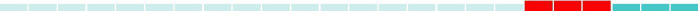
cognitive rehabilitation	2006	2.39	2019	2020	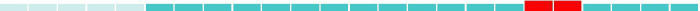
nintendo wii	2020	3.27	2020	2021	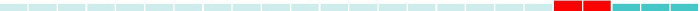
arm function	2016	2.65	2021	2022	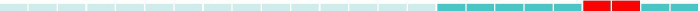
prevalence	2021	2.17	2021	2024	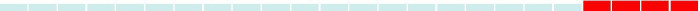
ischemic stroke	2019	3.09	2022	2024	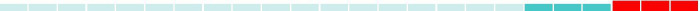
anxiety	2020	2.72	2022	2024	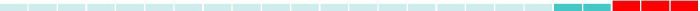
cognitive impairment	2008	2.67	2022	2024	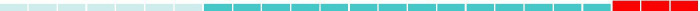
metaanalysis	2022	2.58	2022	2024	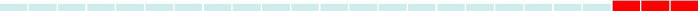
risk factors	2022	2.24	2022	2024	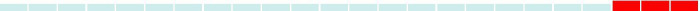
stroke survivors	2022	2.24	2022	2024	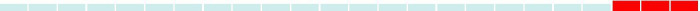

## 4 Discussion

### 4.1 Overall characteristics and development trends in AI-assisted psychological intervention for stroke survivors

Academic publication volume serves as an important indicator for measuring the developmental trajectory of a research field (Zafar and Masood, 2020). Figure 3A illustrates the publication trends in AI-assisted psychological interventions for stroke survivors from 2000 to 2024. The data show that annual scholarly output has demonstrated a steady increasing trend since 2015, peaking at 72 articles in 2022. This growth trend reflects the growing recognition of the importance of AI technology in psychological interventions for stroke survivors. The increasing research interest may be attributed to the potential of AI-assisted psychological interventions to provide personalized assessment, real-time monitoring, and adaptive therapeutic strategies for stroke patients, which can better identify psychological needs and optimize intervention effectiveness, thereby promoting psychological rehabilitation in stroke survivors (Mou and Wong, 2021; Wan et al., 2021; Guillaumier et al., 2022). Meanwhile, the citation analysis in Figure 3B further demonstrates this growth trend, showing significant growth in the field’s scholarly influence. These findings indicate that research on AI-assisted psychological interventions for stroke survivors has attracted continuous scholarly attention, with its scientific value and clinical significance widely acknowledged by researchers.

With the increasing research on AI-assisted psychological interventions for stroke survivors, this study analyzes the global distribution of research outputs by countries, institutions, and authors (Figures 4, 5). Country analysis results show that the United States leads the field with 87 publications, followed by China (76 publications) and Italy (49 publications), while France shows the highest centrality (0.64) (Figure 5B; Table 2). The high research productivity in these countries may be attributed to their advanced research infrastructure, substantial funding support, and well-established interdisciplinary collaboration networks in AI and healthcare research (Hu et al., 2022; Wu et al., 2023). Meanwhile, institutional analysis identifies key research institutions such as McGill University (10 publications), IRCCS Bonino Pulejo (9 publications), and Rutgers University System (7 publications) (Figure 5C; Table 3). McGill University’s leading position may be related to its strong interdisciplinary research programs and the iSMART Lab’s focus on AI healthcare applications (Armanfard, 2024). These institutions provide clear opportunities for academic exchange and collaboration. Additionally, author analysis reveals that Calabro Rocco Salvatore was identified as the most productive author with 9 publications, while co-cited author analysis identifies key academic contributors such as Laver KE and Feigin VL (Figure 4; Table 1). The high productivity of these authors may be attributed to their sustained research focus on stroke rehabilitation and active involvement in AI healthcare research (Li and Sun, 2023). These multi-level analytical results collectively reveal the global research distribution pattern in AI-assisted psychological interventions for stroke survivors, providing a foundation for optimizing resource allocation and international collaboration in this field (Muñoz-Venturelli et al., 2022).

In addition to analyzing countries, institutions, and authors, this study examined journal distribution to further understand the knowledge sources and disciplinary characteristics of AI-assisted psychological interventions for stroke survivors. The analysis reveals that publications in this field are primarily distributed across journals such as the “Journal of NeuroEngineering and Rehabilitation,” “Frontiers in Neurology,” and “Journal of Stroke & Cerebrovascular Diseases” (Table 4), which represent different disciplinary fields including engineering technology, neuroscience, and clinical medicine. This distribution pattern reflects the multidisciplinary nature of AI-assisted psychological interventions, which requires the integration of engineering technology for developing intelligent systems, neuroscientific theoretical guidance to understand psychological changes following stroke, and clinical medical foundations to ensure intervention safety and efficacy. Meanwhile, co-cited journal analysis reveals strong connections between journals such as “Stroke,” “Archives of Physical Medicine and Rehabilitation,” and “Journal of NeuroEngineering and Rehabilitation” (Table 5), further confirming the interdisciplinary knowledge integration between stroke medicine, rehabilitation science, and engineering technology in this field.

Dual-map overlay analysis and reference co-citation analysis further validate the interdisciplinary characteristics identified through journal distribution analysis. Dual-map overlay analysis reveals that literature in neurology, sports and ophthalmology draws significant influence from psychology, education, and social (*z* = 5.22, *f* = 2,194) as well as molecular biology and genetics (*z* = 2.87, *f* = 1,294) (Figure 6). This knowledge flow pattern occurs because AI-assisted psychological interventions cannot rely solely on technological solutions but must be grounded in psychological theories to understand mental health mechanisms, educational principles to design effective intervention protocols, and biological knowledge to comprehend the neurological basis of stroke-related psychological changes (Bajotra and Rani, 2024). Meanwhile, reference co-citation analysis further confirms this interdisciplinary knowledge integration by revealing strong co-citation relationships among foundational studies from different disciplinary backgrounds (Figure 7; Table 6), indicating that the field integrates knowledge from multiple disciplinary sources. Based on these findings, future AI-assisted psychological interventions should enhance interdisciplinary collaboration and strengthen the integration of technological innovation with clinical evidence to improve intervention effectiveness and clinical applicability.

### 4.2 Research hotspot in AI-assisted psychological interventions for stroke survivors

To explore research hotspots in AI-assisted psychological interventions for stroke survivors, this study conducted an analysis of keywords. The keyword co-occurring network map (Figure 1B) and density visualization map (Figure 1C) reveal the research hotspots in this field, with results showing that core keywords such as “stroke,” “rehabilitation,” “quality of life,” and “virtual reality” represent the research hotspots in this area. This keyword distribution reflects an important shift in research focus from traditional rehabilitation approaches to technology-enhanced psychological intervention strategies.

To further understand the developmental trajectory of these research hotspots, this study conducted citation burst analysis to examine the temporal changes in research hotspots (Table 7). During the early period, the emergence of “brain injury” (strength = 2.56) and “traumatic brain injury” (strength = 3.83) reflected research focus on fundamental neurological mechanisms, which occurred because AI-assisted psychological interventions, as an emerging interdisciplinary field, must first be grounded in a deep understanding of neuropathological mechanisms (Bajotra and Rani, 2024). As research progressed, “virtual reality therapy” showed significant emergence (strength = 3.67), with this transition attributed to the maturation of virtual reality technology providing new possibilities for psychological interventions by creating immersive therapeutic environments, thereby effectively enhancing patient engagement and rehabilitation motivation (Zuki et al., 2024). Meanwhile, researchers recognized the need for strict experimental designs to validate the efficacy and safety of these emerging technologies, and “controlled trial” (strength = 3.54) and “randomized controlled trial” (strength = 3.31) subsequently emerged as research hotspot. Additionally, the appearance of “individuals” (strength = 3.69) and “cognitive rehabilitation” (strength = 2.39) reflected deepening clinical understanding that different patients have distinct psychological rehabilitation needs, requiring personalized intervention strategies. The evolution of research hotspots during this period indicates that the field was transitioning from fundamental theoretical exploration toward empirical validation and personalized applications (Ramanarayanan, 2024).

Recent research hotspots show a significant shift toward precision intervention strategies and evidence-based practice. Specifically, the burst strength of “ischemic stroke” (strength = 3.09) indicates that research has begun focusing on specific stroke subtypes. This shift occurs because clinical evidence shows that different stroke types have different psychological rehabilitation characteristics and intervention requirements (Nishat et al., 2021). Therefore, researchers need to develop AI intervention protocols targeted to specific pathological types. Meanwhile, the emergence of “anxiety” (strength = 2.72) and “cognitive impairment” (strength = 2.67) indicates that most stroke survivors simultaneously face dual emotional and cognitive challenges, suggesting that single interventions cannot meet the psychological needs of stroke survivors and comprehensive interventions are needed (Senadheera et al., 2024; Bell et al., 2024). Furthermore, “metaanalysis” (strength = 2.58) reflects the need for systematic evidence synthesis to guide clinical practice once the field has accumulated sufficient evidence (Mitsea et al., 2024). These latest hotspots indicate that AI-assisted psychological intervention research is evolving from technology oriented exploration toward clinically focused, evidence-based development, providing important guidance for implementing personalized and comprehensive psychological interventions in stroke rehabilitation practice.

### 4.3 Clinical application of AI-based psychological interventions

With the evolution of research focus in AI-assisted psychological interventions for stroke survivors from single technology applications toward comprehensive services, and from general interventions toward specific population needs, clinical application strategies also require corresponding adjustments.

First, comprehensive mental health service has become a core component of clinical practice, which aligns directly with the phenomenon of “anxiety” (burst strength 2.72) and “cognitive impairment” (burst strength 2.67) concurrently emerging as research hotspots. In clinical applications, healthcare teams can fully utilize the advantages of AI technology in multimodal data analysis to achieve comprehensive assessment of patients’ cognitive function and emotional states (Arora et al., 2022). Additionally, intelligent systems can identify complex associations between cognitive function and emotional states, providing healthcare professionals with more comprehensive patient status analyses (Sangeetha et al., 2024). Based on these analytical results, clinical interventions can more precisely integrate cognitive training and emotional regulation elements to customize comprehensive intervention plans suited to each patient’s specific needs (Sangeetha et al., 2024).

Second, in the implementation process of comprehensive service, it is essential to strengthen the important role of evidence-based practice in evaluating the effectiveness of AI-assisted psychological interventions, which corresponds with “meta-analysis” (burst strength 2.58) emerging as a recent research hotspot (Nilsen et al., 2024). This requires clinical practitioners to incorporate the latest meta-analysis and systematic review findings when developing guidelines, clearly specifying technology selection criteria, application processes, efficacy evaluation indicators, and quality control points. Such evidence-based clinical intervention protocols can ensure both scientific validity and provide healthcare teams with necessary flexibility in specific practice settings (Majeed and Khan, 2024).

In addition to comprehensive and evidence-based practice, precision intervention strategies represent the third core direction in clinical applications. This trend corresponds with “ischemic stroke” (burst strength 3.09) emerging as a recent research hotspot. Different types of stroke survivors show significant differences in cognitive impairment and mental health needs. This finding suggests that clinical applications require more personalized intervention approaches (Zeng et al., 2021). During the intervention process, healthcare teams can utilize AI technology’s analytical capabilities to develop precision patient classification systems based on stroke type, severity, cognitive function, and mental health characteristics (Shah et al., 2024). Specific intervention protocols can then be developed for different patient groups. This precision medicine approach enhances intervention effectiveness and optimizes healthcare resource allocation, ensuring patients receive psychological health support most appropriate to their condition.

### 4.4 Study strengths and limitations

This study employed bibliometric analysis to examine 450 publications on AI-assisted psychological interventions for stroke survivors published between 2000 and 2024. Using visualization tools such as CiteSpace and VOSviewer, we conducted multi-dimensional analyses of authors, countries, institutions, journals, references, and keywords, revealing research hotspots and development patterns in this interdisciplinary field.

However, this study has several limitations. First, due to technical constraints of CiteSpace and VOSviewer software and the requirement for standardized citation data, we only used the WoSCC database, which may limit the comprehensiveness of our literature coverage. Second, we only included English-language publications, potentially excluding high-quality research published in other languages and limiting the global representativeness of our findings. Third, this study did not differentiate between different AI technologies (such as machine learning, virtual reality, and deep learning) in the analysis, while differences in these technological approaches may exhibit distinct application patterns and development trajectories in psychological interventions. Finally, to analyze the latest research developments, we set the search period until October 2024, and although this included more recent literature, the incomplete data for 2024 may affect the accurate assessment of the most recent research trends.

Despite these limitations, this study contributes to understanding research trends in AI-assisted psychological interventions for stroke survivors. Future research may consider integrating multi-database resources, including literature in multiple languages, and incorporating technological differentiation analysis to increase the representativeness of research findings.

## 5 Conclusion

This bibliometric analysis examined 450 articles on AI-assisted psychological interventions for stroke survivors published between 2000 and 2024. The analysis identified Calabro Rocco Salvatore as the most productive author, with the United States, China, and Italy as primary contributing countries, and McGill University as the leading institution. Journal and reference analysis revealed “Journal of NeuroEngineering and Rehabilitation” as the most productive journal, while the multidisciplinary nature of publications across neurology, rehabilitation medicine, and engineering domains indicates this field requires interdisciplinary integration. Our analysis highlighted several major research hotspots, including ischemic stroke, anxiety, cognitive impairment, and meta-analysis, reflecting the field’s evolution from technology-oriented exploration to evidence-based precision interventions. Future research should develop standardized AI-assisted psychological intervention protocols for different stroke subtypes and enhance interdisciplinary collaboration to promote effective clinical translation.

## Data Availability

The original contributions presented in the study are included in the article/supplementary material, further inquiries can be directed to the corresponding author.
